# Understanding vulnerability and resilience in Somalia

**DOI:** 10.4102/jamba.v12i1.856

**Published:** 2020-12-14

**Authors:** Charles Lwanga-Ntale, Boniface O. Owino

**Affiliations:** 1 Research and Analysis Department, Development Initiatives, Nairobi, Kenya

**Keywords:** vulnerability, resilience, coping, marginalisation, Somalia

## Abstract

In the recent years, Somalia witnessed a heightened frequency of droughts and conflicts. This article explores the experiences of Somalis during the 2011 and 2016 crises, examining the link between vulnerability and resilience, and the role played by international humanitarian responders in resilience building. The aim of this study is to provide information on how different population groups responded to and managed to survive recurrent shocks; the prevailing drivers of marginalisation and exclusion, and mechanisms through which these are maintained; and the role of external stakeholders. A review of literature was combined with field consultations in four study sites: Kismayo Urban, Kismayo Rural, Baidoa and Beledweyne, and complemented by consultations with the Somali diaspora community in Kampala, Uganda. Participatory research methods were used, including participant observation, focus group discussions, household dialogues, livelihood analysis, well-being analysis and gender analysis. The findings of the study revealed an inextricable link between vulnerability, conflict and disasters, with the major challenge facing the most vulnerable Somalis being uncertain about the future. Somali households adopted different coping strategies depending on their resource endowments, including the social and organisational coping strategies, divesting of non-essential domestic assets, and diversification of income generation and food production strategies. Thus, different population groups survived the shocks through social connectedness, which aligned with the effective use of remittances to create robust mechanisms for sharing risk. That notwithstanding, groups that had the backing of more powerful clans seemed to have the edge over those who did not.

## Introduction

Resilience is generating increased attention as an approach for guiding policy response to recurrent droughts, food insecurity and vulnerability in Sub-Saharan Africa (Descheemaeker et al. 2016:2335–2339; Falkenmark & Rockstrom 2008:93–102; Osman-Elasha et al. 2006:27; Shiferaw et al. 2014:5). A heightened frequency of droughts in the last few years in Somalia, where also a major famine in 2011 was followed by near-famine conditions in 2016, has extended research interests towards the possible causal relationships between extreme weather, civil conflict, changing livelihoods and the effect of interventions during the crisis periods (Hillier & Dempsey 2012:5–29; Majid & McDowell 2012:36–42; Maxwell et al. 2016:4).

This is a study on the experiences of Somalis during the 2011 and 2016 crises, exploring the link between vulnerability and resilience, and the role played by international humanitarian responders in resilience building in Somalia. In researching on how different population groups have survived recurrent shocks, this study focuses on the following:

- How different population groups have responded to and managed to survive recurrent shocks
- The prevailing drivers of marginalisation and exclusion, and mechanisms through which these are maintained
- The role of donors, international agencies, non-governmental organisations (NGOs) and other stakeholders in influencing the coping strategies that different communities used.

Three indicative propositions have been made, drawn from the growing body of knowledge on the subject of resilience. Firstly, pastoralist and agro-pastoralist livelihoods in Somalia are increasingly threatened by uncertainties caused by long-term and adverse trends in weather variability and climate change (World Bank & Federal Government of Somalia 2018a:10–150). Rapid deforestation, severe soil erosion, overgrazing and climate change pose significant challenges to the growth prospects and viability of pastoralism and rainfed agriculture (World Bank & Food and Agriculture Organization 2018b:2–30). This is exacerbated by poor infrastructure and low skills, which constrain agricultural production. Secondly and related to the above, violent and non-violent means are being used by segments of the Somali population (primarily leveraging the clan system) to acquire power and to access resources (including land). Somali powerbrokers, local communities, subnational governments and the national government rely, to a great extent, on militias to achieve their interests, including, accessing power, controlling local economies and responding to conditions of insecurity, vulnerability and contestation (Vanda 2020:113–150). This acquisition of power by a few and their increased access to scarce resources (particularly grazing and crop-farming land) can lead to more conflict and loss of life (Menkhaus 2011:1–15). Thirdly, Somalia ranks low in every credible international economic and social index and is defined as a fragile and conflict-affected state (FCAS). Fragile states are often characterised by frequent conflict and insecurity, weak governance and the inability to deliver the efficient and equitable distribution of public goods and services. Weak institutional and governance mechanisms, and the lack of an enabling regulatory and policy environment, present an opportunity for exploitation, routinely leveraged by clans in Somalia as confirmed by this study (Ali, Nicholl & Salzmann 2017:2–12). Thus, legislative and policy decisions may at times safeguard the interests of a few and not the collective. This is the context met by the 2011 famine and 2016 near-famine conditions.

A review of the literature suggests that in Somalia, vulnerability, conflict and disasters are closely interlinked and the major challenge facing the most vulnerable people is uncertainty about household and community sustainability (Langworthy et al. 2016:1–90; Teshome 2011:55). For example, long-term trends such as environmental degradation and high population growth undermine household resilience and create uncertainties about the future viability of livelihood activities such as pastoralism (Menkhaus 2003:50; World Bank & Food and Agriculture Organisation 2018b:33–65). In addition, governance in Somalia is characterised by weak traditional and formal polities with limited ability to provide basic services and security (Menkhaus 2006:74–106, 2014:154–172; Zeinab, Samantha & Zach 2017:10–11). The literature also highlights the persistence of gender disparities in the country that has over the years influenced household resilience and access to, ownership of, and control over assets. A key observation from the review of literature is that during the two crises, Somali households adopted different coping strategies depending on their resource endowments. The strategies included migration, livelihood diversification, borrowing money, reliance on external support, crop rotation, mixed cropping, an adaptive planting calendar in response to weather patterns and harvesting run-off water for irrigation.

Regarding the role of external actors, the literature highlights three key features, namely, social networks, safety nets to support individuals and households during the two crises (e.g. the Unconditional Cash and Voucher Response implemented during the period 2011–2012) and remittances sent by the Somali diaspora community (Hedlund et al. 2015:1–20; Truelove & Duncalf 2012:6–11). Remittances to Somalia, estimated at US$1.2 bn annually (Food Security and Nutrition Analysis Unit 2013:1), promoted household resilience by facilitating access to food and services such as healthcare, as well as foreign exchange for importing essentials such as foodstuffs, even though they reached only about 20% of Somali households (World Bank 2017:35). Households also relied on social networks to promote resilience. They achieved this by allowing communities to pool resources together to strengthen their livelihoods and respond to shocks (Majid, Abdirahman & Hassan 2017:15–33). According to Mercy Corps and Tango (2013:5), during the 2011 Somalia famine, households that had greater inter-clan social and economic interactions were less food insecure as they could access assistance within their clan networks.

## Methodology

### Analytical framework

In Somalia, poverty, vulnerability, conflict and disasters are closely interlinked and, therefore, have to be understood in an integrated manner based on approaches that unify different yet complementary analytical frameworks. Accordingly, this study employed Practical Action’s Vulnerability to Resilience (V2R) framework that draws on several frameworks, including the sustainable livelihoods framework, disaster management and climate change adaptation, combining these into one integrated framework. The V2R facilitates qualitative analysis of vulnerability and resilience through a participatory approach that involves vulnerable people themselves as active research designers and participants that inform the understanding of vulnerability from the community perspective, its root causes and the most affected groups.

**FIGURE 1:**
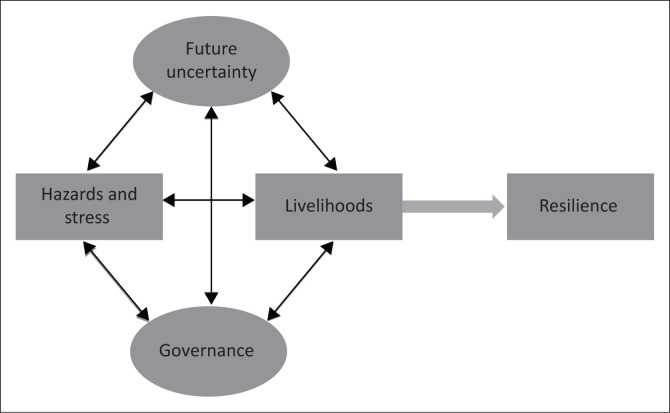
The vulnerability to resilience framework.

The V2R framework links household resilience to four interrelated factors that influence vulnerability, namely, exposure to hazards and stresses, fragile livelihoods, future uncertainty and governance (Figure 1). Vulnerability refers to the ‘degree to which a population or system is susceptible to, and unable to cope with hazards and stress, including the effects of climate change’ (Pasteur 2011:11). A number of factors contribute to the vulnerability of a population, including physical exposure to hazards, and social and economic conditions that affect access to livelihoods, such as lack of savings and poor education. For instance, a community can be vulnerable to flooding if its members are living along unprotected river banks. Livelihood conditions often influence physical exposure to hazards. For instance, poor households may be forced to live and work in unsafe locations such as informal settlements with inadequate water and sanitation facilities because their options are limited. Fragile resources can be damaged easily when a disaster strikes, making it difficult for households with little or no savings to cope with and to recover effectively. Vulnerability can also be exacerbated by the uncertainties stemming from long-term trends such as climate change and population growth, which are often not understood adequately by poor households. Moreover, lack of a voice and inclusion in the policy and decision-making processes that influence access to resources may limit the ability of households to address the underlying causes of their vulnerability.

Resilience refers to the:

[*A*]bility of a system, community or society to resist, absorb, cope with and recover from the effects of hazards and adapt to longer term changes in a timely and efficient manner without undermining food security or wellbeing. (Pasteur 2011:13)

Resilient households have the capacity to endure shocks and to recover from and adapt their livelihoods when affected with hazard events. Strengthening household resilience, therefore, calls for measures aimed at addressing the root causes of vulnerability (Figure 1). In particular, supporting households to establish diversified and secure livelihoods can enhance their resilience by improving their ability to live and work in locations that are less exposed to hazards and increasing the resources they can draw on to cope with and recover from shocks. Households can significantly reduce their exposure to negative impacts if they are adequately prepared for hazard events. Furthermore, adequate understanding of long-term trends, especially those that affect livelihoods and well-being such as climate change, can enable households to use their resources in ways that promote adaptation over time. An enabling governance environment is also important for building resilience as it can enhance access to basic services, equitable allocation of resources and inclusive policy and decision-making processes.

The research framework for this study was anchored on three key concepts of risk, vulnerability and resilience and focused on the key research question: ‘[*h*]ow have different population groups in Somalia survived recurrent shocks’? To tackle this question, we examined the different categories of hazards and stresses that faced vulnerable Somalis during the 2011 and 2016 crises, exploring the differences in each period and how livelihoods changed during this time. Furthermore, we examined how different individuals, households and communities tackled multiple hazards, especially given the protracted impacts of conflict, climate change and chronic poverty, and in light of the uncertainties faced and the local coping strategies used. Ideas were then developed on how the positive adaptations made by communities could inform future resilience policy and programming. Based on an interactive analysis of information in (and between) each of the above clusters (i.e. hazards and stresses, future uncertainty, livelihoods and governance), we concluded the link between vulnerability and resilience, and the role played by international humanitarian responders in resilience building. Lessons were also drawn on how the coping strategies had led to new practices and enabled some population groups to not only survive but also thrive, despite the recurrent shocks.

### Approach and methods

A detailed review of published and grey literature preceded fieldwork and focused on risk, vulnerability and resilience during crises in the study period. Field consultations were carried out in four study sites: Kismayo Urban, Kismayo Rural, Baidoa and Beledweyne. These sites offered diverse livelihoods and diverse experiences of risk, vulnerability and resilience. The sites were also the most representative in terms of having major agro-ecological zones in close proximity, the presence of humanitarian actors, and a history of drought and its resulting food insecurity, conflict, insecurity and inaccessibility.

The study employed a wide range of participatory research methods, including participant observation, focus group discussions (FGDs), household dialogues, livelihood analysis, well-being analysis and gender analysis (Chambers 1992:15–17). Following the field consultations, data were analysed using a deductive analytical approach. Information from transcribed daily reports was categorised by subject and topics based on predetermined research questions. Categorised information was then assigned to each research objective before being further summarised into emerging patterns, validated and assessed.

Stratified random sampling was used to identify and select respondents in the study sites. This strategy was used to ensure inclusion and participation of all groups (youth, older persons, women, minority clans and persons with disabilities) in the study. Key informant interviews with representatives of local and international NGOs, government officials and clan elders were also included. The key informants were selected based on the purposive sampling strategy. A total of 256 respondents were interviewed, that is, 108 in Kismayo (urban and rural), 68 in Beledweyne and 66 in Baidoa in Somalia, as well as 14 respondents living in Kampala, Uganda. These included women, youth, elderly people, persons with disabilities and internally displaced persons (IDPs); they were interviewed both in groups and as key informants.

Study limitations included a short research timeframe; local researchers’ limited expertise; difficulties in translating technical terms into local languages; caution by government officials, Al-Shabaab (AS) operatives and sympathisers regarding the research; insecurity in AS-controlled areas and cultural factors driving gender exclusion and clanship. To mitigate these challenges, we worked with local field researchers with in-depth knowledge of local dialects, the study areas and local culture.

### Ethical consideration

The researchers gained informed consent for interviews from all the participants. Additionally, the participants were given the option to opt-out at any time. All the participants interviewed in this study were legal adults and were able to consent to their own participation. The relevant government authorities in Somalia were also informed about the study.

## Results and discussion

The findings reveal an inextricable link between vulnerability, conflict and disasters, with the major challenge facing vulnerable Somalis being uncertainty about the future. In 2011 and 2016, the main hazards in Somalia were a combination of drought and hydro-meteorological risks. However, the hazards were handled differently in 2011 and 2016. Households adopted different coping strategies depending on their resource endowments. These included social and organisational coping strategies, divesting of non-essential domestic assets; and diversification of income generation and food production strategies. Marginalisation and exclusion of minority clans, women and persons with disabilities in access to resources and participation in decision-making processes continue to undermine efforts to build household resilience in Somalia. In 2011 and 2016, external stakeholders, including donors and international non-governmental organisations (INGOs) influenced the coping strategies that were adopted by the affected population through a number of measures, including the provision of relief, skills development and promoting income diversification.

### How different population groups managed to survive recurrent shocks

#### Hazards and Stresses: In what ways was 2016 different from the 2011 crisis?

Susceptibility to drought was itself blamed on factors ranging from weather variability to poor land use, exacerbated by uncontrolled deforestation to produce charcoal for domestic use and for export. Recurrent droughts have led to persistent food insecurity, especially amongst households with young children as stated by a female respondent in rural Kismayo:

‘These are extremely difficult times for most households, especially those with children. Hunger is a daily struggle because our people can no longer rely purely on their cattle due to recurrent droughts and in an environment where the rains are sporadic and the soils are poor, growing crops remains a significant challenge.’ (Participant 15, middle aged, female, villager)

Hazards and stresses were, however, handled differently in 2016, compared with 2011. After the 2011 famine, there was a significant increase in drought mitigation and disaster planning efforts. At regional level, the inter-Governmental Authority for Development developed the Drought Disaster Resilience and Sustainability Initiative strategy for the region to promote more sustainable and holistic drought mitigation approaches. Similarly, the Somali government, working in partnership with donors, and humanitarian and development actors, made improvements to the country’s early warning system; developed more collaborative disaster risk management plans and scaled up the implementation of social protection programmes. Thus, when the disaster struck in 2016, most people were better prepared and had several mitigation measures in place.

In addition, local communities were better reached by social protection interventions, remittance flows and shared family resources. However, not all hazards and stresses could be effectively dealt with in 2016. For example, deforestation and bush fires related to the proliferation of charcoal production and trade remained problematic, and the prevalence of human immunodeficiency virus (HIV) and acquired immunodeficiency syndrome (AIDS) exacerbated health risks.

#### Livelihoods: How did they change?

Livelihoods in 2016 differed considerably from those in 2011. Whilst several factors were responsible for Somalia’s famine in 2011, weak governance exhibited by the Transitional Government and accompanying corruption, political instability and an unending insurgency were at the forefront of the challenges. These led to a slow response to the famine, inefficiencies in planning, coordination and forecasting of the aid response, and ineffective planning for future crises. In contrast, 2016–2017 saw more, faster and better-coordinated humanitarian assistance because of existing personnel in most areas, up-to-date and collaborative Disaster Risk Management strategies, and more prompt responses by donors and NGOs. However, access to humanitarian assistance remained a challenge to vulnerable groups, such as the elderly, children and persons with disabilities, who were not able to travel to aid distribution centres in urban areas or IDP camps. This is illustrated by a response from a person with disability in Baidoa who stated that:

‘During the 2016 drought, I was unable to migrate to urban areas to seek assistance since I was not able to meet the transportation costs and I was not able to walk. My people left me in the village, desperate and hungry. I suffered from acute malnutrition until a good Samaritan rescued me and took me to an IDP camp in Baidoa.’ (Participant 92, elderly, male person with disability)

Furthermore, the 2011 famine experience had led to households keeping dry rations ‘for difficult times’ whilst also diversifying their source of income. In the intervening period between 2011 and 2016, various development agencies and NGOs had worked with communities to start small businesses, thereby contributing to more employment opportunities. Similarly, the 2011 famine occurred during a period of heightened insecurity, and this significantly had an impact on people’s livelihoods. For example, Kismayo and Juba were under the control of AS who restricted access to the area by humanitarian organisations. Thus, the affected communities received very limited relief aid, and venturing afar for food was risky. In addition, skills training was scaled up post-2011 to diversify incomes. For example, vocational training centres were established for young people to acquire practical skills for self-employment in the informal sector. Vocational training enabled some households to build resilience by engaging in self-employment to improve their income, as illustrated by a male respondent in Baidoa:

‘I was an IDP with five children and was previously selling ice cream for a living. However, after completing a tailoring course at a local vocational training institute which was funded by the Danish Refugee Council, I started a tailoring business and am now able to support my family.’ (Participant 113, middle aged, male, tailor)

Apart from vocational training, some households, especially those that stuck to agro-pastoralism as their main form of livelihood, adopted drought-tolerant crop varieties. Similarly, working mostly with NGOs, farmers were better organised, enabling them to share information and better articulate their needs.

The use of cash transfers and remittances was another difference between livelihood activities in 2011 and 2016. In 2011, the two financial instruments were limited and uncoordinated but became key in ensuring access to food and in the transformation of livelihoods during the 2016 crisis. Unconditional transfers made in regular monthly instalments enabled households to purchase foodstuffs, start small-scale businesses and to invest in asset building (e.g. education and health). In Baidoa, for instance, a respondent stated that:

‘I was previously working at a construction site as a labourer. However, when unconditional cash transfer programmes were launched during the crisis, I received $750 and used part of it to start a small clothes shop. Now am able to support my family including paying school fees for my children despite the absence of my husband who left the country to seek asylum.’ (Participant 78, middle aged, female small-scale business person)

In line with the cultural tradition of sharing, recipients shared their income from both remittances and cash transfers with neighbours and friends, or in the case of urban recipients, with other family members who may have remained in the rural countryside.

Finally, during the 2016 crisis, the Somali government, working in collaboration with the United Nations, sought to curb charcoal production. Before 2011, charcoal trade was a means of livelihood, especially for young men, but it was blamed for environmental degradation in Somalia. Hence, state and non-state actors called for policies and programmes to reduce charcoal production, and to provide livelihood and energy alternatives to the affected people. A key difference noted is that in 2016, the Somali government gave stronger and better-coordinated leadership than it did in 2011, leading, for instance, to the Joint Programme for Sustainable Charcoal Reduction and Alternative Livelihoods.

#### Future uncertainty: What are the long-term trends?

The two Somali crises of 2011 and 2016 highlighted the urgency and difficulties that are associated with addressing future uncertainty in the process of building resilience. Firstly, evidence from the field and a review of literature confirmed the connectedness between weather variability, environmental degradation and conflict on the one hand, and declining livelihoods and food insecurity on the other hand. Data limitations in the study hampered similar linking of trends in urbanisation, HIV and AIDS and climate change. On weather predictability, the establishment of a robust early warning system for Somalia now ensures that better information is generated and made available to and is used by communities.

Despite the observations above, it is perhaps regarding major long-term changes in types of livelihoods that future uncertainty should be further examined. Pure pastoralism (i.e. camels, cattle, goats and sheep), agro-pastoralism and fishing are the primary livelihoods for most rural communities in Somalia, and pastoralist and agro-pastoralist livelihoods are recurrently affected by drought. Limited land and water resources, coupled with ongoing environmental degradation and movement restrictions for grazing animals (because of fencing of land in some areas), pose additional challenges. Pastoralist livelihoods are currently under siege in Somalia. Recurrent droughts, the progressive disappearance of what used to be communal grazing lands and the fragmentation of rangelands into new agricultural settlements have combined to weaken customary institutions that previously sustained animal wealth in the region. Action will, thus, be needed to ensure that those who wish to remain pastoralists are supported to find alternative or complementary ways of maintaining this type of livelihood.

### Governance

For close to two decades, Somalia has had no effective government. Instead, various systems of governance at national and local levels evolved, including those based on ethnic, clan or military lines, some providing modest levels of security and the rule of law. During 2011, the multiplicity of uncoordinated governance arrangements, weak institutional mechanisms, and absence of an enabling regulatory framework and environment negatively affected policy-making, investment, trade and service delivery. This situation changed significantly in 2012 (1 year after the devastating famine of 2011), beginning with the passing of the country’s provisional constitution and formation of the Federal Government of Somalia (FGS). Since then and because of improved governance, Somalia made significant progress towards realising political, security and economic development. By the time the 2016 drought occurred, institutions were in place and systems for regular planning and monitoring, for instance, were already functioning. Specifically, establishment of the Ministries of Agriculture, Environment and Livestock, and of the Humanitarian Affairs and Disaster Management Agency (HADMA) transformed the way priorities were being made to address emerging crises in the country.

### Strategies for coping and building resilience

The concept of resilience has gained popularity amongst donors and other development actors in relation to disaster risk management. The key objective underpinning the concept’s current popularity is the switch of focus from repeatedly providing humanitarian assistance, in addition to, enhancing the capacities of individuals, households and communities to resist shocks and manage impacts on their own, whilst maintaining or transforming their own living standards. In this article, we observed that a number of coping strategies were also used to promote or build resilience.

Three broad categories of coping strategies emerged during the 2011 famine and 2016 near-famine crises, namely, (1) social and organisational coping strategies, (2) divesting of non-essential domestic assets and (3) diversification of income generation and food production strategies.

The most prevalent category of coping strategies, especially during the 2011 famine, was the social and organisational type. This is akin to the social connectedness category espoused by Maxwell et al. (2016:8). Closer social connections were observed amongst families, friends, gender and youth groups, clan members, acquaintances, professional groupings and even those connected through religious alliances. The connections primarily operated on a reciprocal basis, which enabled participating group members to support each other during crises. Through various social networks, links were regularly established between communities and authorities, who provided food, medical or other forms of social assistance. However, these networks were more complex than the categories provided may suggest. Although the characteristics of the membership of these networks spanned geography, age, gender and religion, sharing these and others (such as belonging to the same clan or originating from the same neighbourhood) did not necessarily mean that people converged to form a network. More insights into the determinants of convergence around certain characteristics need to be obtained. Nonetheless, social networks were reported to have played a vital role in influencing the decisions that IDPs made about a wide range of survival and livelihood issues. For example, they advised on when people should leave their home areas and where they should go. Even when IDPs arrived in the destination areas, their social networks offered information and advice on where to find accommodation and food, how to navigate the new environment and where to register as an IDP, if needed. Furthermore, these networks provided emotional support to IDPs, an additional coping strategy.

Divestment and disposal of non-essential assets were another popular category of coping strategies, especially during the 2011 famine. This strategy involved distress sales and success depended largely on the diversity of assets owned by the household. On the one hand, smaller stock such as goats, chicken and sheep were sold first, followed by young cattle and as a last resort milking cows, working oxen and camels. Given that most stocks were already deteriorating because of lack of pasture, such sales mostly attracted less than their original value. On the other hand, the value of inanimate assets such as jewellery, bicycles, building materials and electronic materials (including radios) remained stable. In all instances, the decision to sell was influenced mostly by the range of assets a household had, the known or perceived utility of the items being offered for sale and the urgency of needs within the household.

The diversification of income generating activities and food production, another coping strategy, was more evident in 2016–2017 and included charcoal burning, hunting and gathering (especially for those in rural areas), handicraft production, paid domestic labour and engagement in petty trade in urban areas. Some families also engaged in livestock trading to generate money for food and to relieve their households of non-productive animal stocks. As a strategy for coping and risk management, therefore, such diversification involved the combination of the following options, at times all at once: production activities on their own farm, wage labour earned from others’ farms or engagement in other non-farm works. In nearly all instances, this type of diversification was survivalled and had limited returns.

### Drivers of marginalisation and exclusion

Agro-pastoralist livelihoods in Somalia have for centuries been supported by traditional land tenure systems that promoted free access to pasture and water. However, over the years, reduced mobility because of insecurity and the emergence of state and private land ownership have restricted access rights. This has led to an unequal distribution of land and access to pasture and water, leading to marginalisation of minority communities with limited influence in distribution of resources.

Marginalisation and exclusion in Somalia are also perpetuated through traditional clan identities. Clan structures are used by local authorities, including clan elders, to allocate resources and influence access to power, leading to marginalisation and exclusion of minority clans. A key informant stated that ‘the narrative amongst the ruling clans in Somalia is that there are no ethnic or religious minorities in the country’. The dominant clans use this claim to retain power and to continue to shape political and resource allocation decisions by creating the perception that Somalia is a homogenous nation where there are no marginalised minorities.

Power sharing amongst the clans in the country is based on a system that institutionalises marginalisation and exclusion of minority clans. Political power is shared based on the 4.5 formula – an arrangement agreed at the Arta Somalia National Peace Conference in 2000. The 4.5 formula allocates equal stakes in government to the four main clans, whilst the minority clans together have to share the remaining 0.5 stake. Accordingly, the minority are under-represented and lack a voice in decision-making processes.

The culture of patriarchy and gender discrimination also contribute to marginalisation of women. The gendered division of labour continues to limit the participation of women in mainstream socio-economic activities, leading to gender inequalities in the county. Despite the efforts that have been made by NGOs to eradicate gender inequalities, the status of women in Somalia remains subservient with defined roles based on customary laws that are specific to each clan.

The protracted violent conflicts in the country have had significant negative impacts on the physical and mental health of men and women in the country. Accordingly, women had to take up roles that were traditionally performed by men, such as being the primary earner in their households. This is illustrated by this excerpt from an interview in Kismaiyo:

‘After the war and political crisis started, in most households, women have become the primary earners while men have become less economically active due to stress, substance abuse (particularly khat), injuries and inability to find socially appropriate livelihood opportunities.’ (Participant 46, elderly, male, pastoralist)

Whilst women have to work to support their families, they have little or no right to make decisions on access, ownership and use of resources at the household and community level, limiting their capacity to participate socially and economically in their communities.

Limited access to education, especially amongst women and girls is also a key driver of marginalisation. Denying or limiting educational access to social groups that are already disadvantaged such as persons with disabilities and women perpetuate marginalisation and exclusion by limiting the ability of such groups to participate in political and decision-making processes, which, in turn, lead to disempowerment and disenfranchisement.

### Role of external actors in influencing coping strategies

External stakeholders played a key role in supporting households and communities to cope with the 2011 and 2016 crises. Cash transfer programmes were the major investments that were made by INGOs in order to enable households to cope with the crises and to strengthen their resilience to future shocks. In Beledweyne, for instance, a female participant in a FGD indicated that cash assistance received from INGOs enabled many households to access food, prevented further displacement of communities, and strengthened the capacity of households to recover and to rehabilitate their livelihoods after the crises. Programmes, such as the Empowering Entrepreneurs with Skills through Income Generation Activities, implemented by Save the Children were reported by participants in FGDs to have enabled vulnerable households in IDP camps to diversify their income and to improve their food security.

Remittances from the diaspora were not only important for coping with the crises but also contributed to resilience through income diversification activities, such as starting small businesses. Additionally, some communities in the study sites contributed part of the remittances to their communities to support infrastructure development initiatives, such as rehabilitation of water facilities. External stakeholders, particularly the World Bank in 2016, collaborated with the Federal Government of Somalia to ensure continued access to remittances, after the closure of bank accounts of Somali-owned money transfer companies in the United States and Europe because of money laundering risks (World Bank 2018:1–33). This included supporting the Central Bank of Somalia to develop mechanisms for regulating and supervising money transfer businesses in the country in order to prevent money laundering.

External actors, including regional and internal media, donors and INGOs, were also instrumental in raising awareness about the crises and supported efforts to mobilise resources that were needed to provide relief to affected communities. A key change observed in the involvement of external actors in the 2011 and 2016 period is the gradual shift from being providers of relief to facilitators of planning and institutional-building initiatives. Such shifts are expected to strengthen resilience through better disaster preparedness and improved adaptive capacity.

Whilst the aid delivered by external actors was fundamental for coping with the crises, its distribution faced significant challenges. Most INGOs focused on delivering assistance in urban areas where accessibility and security were better than rural areas. This led to an influx of people in urban areas and IDP camps that had inadequate social amenities and infrastructure, leading to tensions between the host and the displaced communities. Furthermore, the reliance on the clan structure to distribute aid prevented transparent delivery of assistance, thereby perpetuating the exclusion of vulnerable groups. For instance, interviews with persons with disabilities showed that most of them were not able to access aid distribution centres because of mobility challenges, whilst those who received assistance often lost it to militias through taxation or to their caregivers who diverted the aid to their personal use. In Baidoa, for instance, a participant in FGD with persons with disabilities stated that:

‘Some non-governmental organizations do not recognize us as a group with special needs as they focus on providing aid to people in IDP camps which we are not able to access.’ (Participant 53, male, person with disability)

This underscores the importance of strengthening the targeting processes to ensure aid reaches the most vulnerable people.

Another challenge that was reported by key informants working for INGOs and local governments was duplication of efforts in provision of assistance. This included the implementation of different cash transfer programmes that targeted the same communities, but lacked effective coordination or harmonisation. Furthermore, the commitment of INGOs to pass the ownership and management of aid delivery to local organisations was questioned by key informants who stated that INGOs were not implementing adequate capacity-building programmes to equip their local partner NGOs with the expertise and skills needed to manage the delivery of aid.

## Conclusion

This study analysed the experiences of Somalis during the 2011 and 2016 crises to provide insights on why some Somali communities have been able to survive recurrent shocks, whilst others have remained vulnerable and marginalised. To this end, the study explored how different population groups responded to and managed to survive the 2011 and 2016 crises, the prevailing drivers of marginalisation and exclusion, and the roles of external stakeholders in influencing the coping strategies that different communities used.

Overall, the findings show that three broad factors enabled Somali communities to survive recurrent shocks. The first is that in 2011, large numbers of people (estimated to be over 250 000) lost their lives, and the quality of life for those who survived declined considerably. In the 2016 drought, however, a few complementary factors converged. Social connectedness aligned with the effective use of remittances to create robust community mechanisms for sharing risk. However, those who had the backing of more powerful clans or ethnic groups seemed to have an edge over those who did not or who belonged to weaker clans. Similarly, individuals and households not only diversified their income sources but also developed new skills for earning livelihoods. Furthermore, in the period leading up to the 2016 drought, investments in social protection mechanisms paid off and cash transfer instruments were better leveraged because of improved coordination and provision of complementary services.

The frequency, magnitude and complexity of disasters in Somalia, coupled with vulnerability and the lack of preparedness on the part of local communities, highlight a few issues for policymakers and other development actors. Better climate policies are needed within Somalia and beyond. In relation, and because of the recurrent and prolonged nature of drought and conflict in Somalia, it is urgent that a more holistic and comprehensive approach to addressing disasters in the country is adopted – one that seeks to address the underlying causes of hunger and poverty in the country, concurrently responding to food and livelihood insecurity, whilst also addressing such factors as conflict, public health concerns (including HIV and AIDS), rapid population growth, environmental degradation and governance.

Several long-term trends undermine household resilience, including climate change, high population growth, environmental degradation and conflict. Future uncertainty, however, is of highest concern for Somalis. In 2011, a considerable gap was pointed out between the perspectives held by professionals who were providing early warning information on the then worsening drought, and donors and the humanitarian community who wanted more concrete information to substantiate the nature, magnitude and complexity of the drought, thus wasting time leading to delayed responses. The failure on the part of the development community to act on the information provided by scientists and NGOs highlighted a gap in communication and decision-making that led to massive loss of life. The government of Somalia should work with development partners to link early warning systems, food security agencies and social protection programmes to enhance the efficacy of assistance provided. This calls for enhancing the capacity of the government of Somalia to play its strategic leadership and coordination roles, programme implementation and policy coherence, and promotion of suitable climate-sensitive livelihood options and related new skills.

Finally, the crises that Somalia experienced in 2011 and 2016 ought to be viewed through the lenses of a changed and changing landscape – politically, demographically, socially, environmentally and economically. Drivers in each of these key clusters have played a role (and continue to do so) in livelihood outcomes, vulnerability and resilience. Livelihoods such as pastoralism or agro-pastoralism are being eroded by such protracted crises. Better coping strategies and alternative livelihood options are needed – despite continued challenges of dwindling pastures, limited access to water, proliferation of small arms and weak policy interventions. This calls for collaborative efforts that bring together the government of Somalia, academia and development partners to explore what works or does not on resilience and newly used coping strategies and livelihood options, documenting lessons for policy and programme uptake. This should include exploring how best to draw on community knowledge – on their use of social capital in conflict and drought conditions.
